# Vectorized building rooftop prints of the Qinghai-Tibetan Plateau and its neighboring regions

**DOI:** 10.1038/s41597-025-05266-4

**Published:** 2025-06-17

**Authors:** Tao Ye, Hongyu Shan, Jidong Wu, Qiang Zhou, Mingfu Ma, Wenzhi Zhao, Ru Ya, Yuan Gao, Lizheng Wu

**Affiliations:** 1 State Key Laboratory of Earth Surface Processes and Disaster Risk Reduction, Beijing Normal University, Beijing, 100875 China; 2 Key Laboratory of Environmental Change and Natural Disasters, Ministry of Education, Beijing Normal University, Beijing, 100875 China; 3 Academy of Disaster Reduction and Emergency Management, Ministry of Emergency Management and Ministry of Education, Beijing, 100875 China; 4 Faculty of Geographical Science, Beijing Normal University, Beijing, 100875 China; 5 School of National Safety and Emergency Management, Beijing Normal University, Beijing, 100875 China; 6 School of Geographic Science, Qinghai Normal University, Xining, 810016 China; 7 Academy of Plateau Science and Sustainability, People’s Government of Qinghai Province and Beijing Normal University, Xining, 810008 China; 8 School of National Safety and Emergency Management, Qinghai Normal University, Xining, 810016 China; 9 Alibaba DAMO Academy, Shanghai, 200032 China

**Keywords:** Geography, Environmental economics

## Abstract

Large-scale high-precision building distribution data is important fundation for regional urban planning and resource allocation and disaster risk research. The Qinghai-Tibetan Plateau is the third pole of the world. Although understanding local human–environment interactions in the Qinghai-Tibetan Plateau is critically important, this has been hindered by a lack of high-resolution building footprint data due to the vastness and remoteness of the area. In this study, we generated the first vectorized building rooftop prints of the Qinghai-Tibetan Plateau and its surrounding areas by using high-resolution Google imagery and the building contour extraction algorithm of the AI Earth platform. Our results include 13.09 million buildings covering 6092.7 km^2^, validated with a total of 250 × 1 km^2^ test samples. The data had an overall accuracy of 87%, a recall of 91.9%, and an F1 score of 64.8%, thus providing an advanced description of the building distribution of the study area as compared to CBRA. Our work has immense potential in facilitating exposure assessment for studies on disaster risk in this area.

## Background & Summary

The advent of the digital era has increased the demand for reliable data on building distribution and attributes^1–3^. Building distribution data provide important spatial information of not only buildings but also population and physical assets^4^, serving as a good proxy for human activity. In recent decades, building distribution data have been widely used in monitoring urban and rural development^5,6^, understanding the impacts of urbanization on food security, biodiversity, climate change, and public well-being and health^7,8^, formulating regional development strategies, and protecting urban and rural ecosystems^9–11^.

The advancement of satellite-based and airborne imagery, together with recent progress in machine learning and deep learning algorithms, has boosted the availability of building distribution data^12–14^. Building distribution data have been provided in raster format as a part of land use/cover data. The most up-to-date release includes three 10-m global land use/cover products based on Sentinel satellites, namely Google’s Dynamic World (DW)^15^, Esri’s 2020 Land Cover^16^, and World Cover 2020 (WC) of the European Space Agency (ESA)^17^. Besides spatial distribution information, researchers are also trying to attach attribute information to building pixels, such as building height^14^. For example, He *et al*.^7^ used multi-source remote sensing data fusion to construct the world’s first 30-m-resolution urban three-dimensional spatial and temporal sprawl dataset covering the period from 1990 to 2010. Despite the continuous improvement of spatial resolution, raster-based building distribution data still cannot describe spatial objects^18^, and increasing resolution greatly increases storage and computing costs^12^.

Building distribution data in vector format, also known as vectorized building rooftop or building footprints, are the outline data of a building projected onto the ground in an overhead view^19,20^, which provide information such as the geographic location, spatial extent of the boundaries, and footprint of a single building. Public service providers (e.g., Google Earth and OpenStreetMap) provide open-access vectorized building rooftop data with wide coverage, fast updates, and low cost^21–23^. In 2022, Microsoft Corporation used deep neural network–based semantic segmentation to extract the outlines of 777 million buildings, some of which containing building height attributes, based on Bing Maps (including Maxar and Airbus imagery) from 2014 to 2021 for every continent outside of China. On May 30, 2023, Google released a dataset of 1.8 billion building outlines extracted from 0.5-m high-resolution satellite imagery, covering an area of 58 million km^2^. The low redundancy and compact structure of vector data represented by vertices and paths provide higher geographic accuracy independent of mesh size, and the introduction of topological rules further improves the integrity of vector data^13^.

Due to the increase in imagery resolution and the decrease in data acquisition costs, together with recent progress in extraction algorithms, the resolution and coverage of building distribution data are continuously improving^24^. The viability of very-high-resolution (VHR) images has enabled the extraction of building footprints by the application of traditional hand-crafted feature-based methods^25^ or deep learning–based methods^13^. As the former approach faces the challenge of diversity of building appearances and sizes, and complex rules of thumb and threshold settings, it is limited when applied to large-scale high-resolution remote sensing images^26^. Deep learning–based methods (e.g., convolutional neural networks) have shown effective and superior performance in automatically learning high-level and discriminative features in building scene segmentation. Sun *et al*.^27^ proposed a fusion strategy based on parallel support vector machines to fully utilize deep features extracted from multi-scale convolutional neural network structures at different scales, with superior performance in extracting complex buildings in urban areas. Nevertheless, when segmenting buildings, the accuracy of the model is more likely to be constrained by the quality of the training samples, making extrapolation difficult. Insufficient use of high-level semantics and omission of low-level details in deep models, resulting in edge-blurring and small-building omissions, also hinder the application of deep learning in building footprints extraction.

The Qinghai-Tibetan Plateau region is the world’s most elevated area, with an average elevation of >4000 meters above sea level, and covers an area of 2.5 million km^2^ ^28^. More than 10 million people inhabit the region despite its extreme climate, cold and long winters, large annual and diurnal temperature differences, and poor indoor thermal environments. Although this region is the largest ecological barrier in China^29^, human activity considerably impacted its vulnerable eco-environment^30^. This region is also extremely disaster-prone, with earthquakes, landslides, mudslides, glacial lake outburst floods, and snow disasters leading to casualty and property losses^31–33^. In response, accurate building distribution data are critical for modelling human activity distribution for coupled human–environment study^34,35^, as well as exposure and risk analysis for natural disasters^36,37^. In addition, this region has long sunshine hours, abundant solar energy resources, and sufficient solar energy collection surfaces such as rooftops and open spaces^38,39^. High-resolution rural building distribution data could provide a reliable database for evaluating photovoltaic potential and efficiently improving the living standards of those living in rural areas^40,41^.

As an underdeveloped region, the Qinghai-Tibetan Plateau and its neighboring areas still do not have a complete set of high-precision vectorized building rooftop data, owing to their vast area, sparse building distribution, remote location, and resource constraints. The most ready-to-use data are those provided at the national scale of China, which include the 2.5-m gridded China Building Rooftop Area data (CBRA, Liu *et al*., 2023b) and China’s first national land cover map with 1-m resolution (SinoLC) that includes building categories^42^. These raster data are unable to characterize spatial objects and require large storage resources. The vectorized rooftop area data for 90 major cities in China released by Zhang *et al*.^13^ partly filled this gap; however, only 14 cities in the Qinghai-Tibetan Plateau were included in this dataset, and vectorized building rooftop data are still absent for an area of 2 million km^2^.

Therefore, this study aims to generate vectorized building rooftop prints of the Qinghai-Tibetan Plateau and its neighboring regions by incorporating high-resolution satellite imagery and deep learning algorithm. Our dataset was validated using test samples comprising 250 × 1 km^2^ grids across various sub-regions, resulting in an overall accuracy of 91.92% and an F1 score of 64.81%.

## Methods

### Framework

**Fig. 1:**
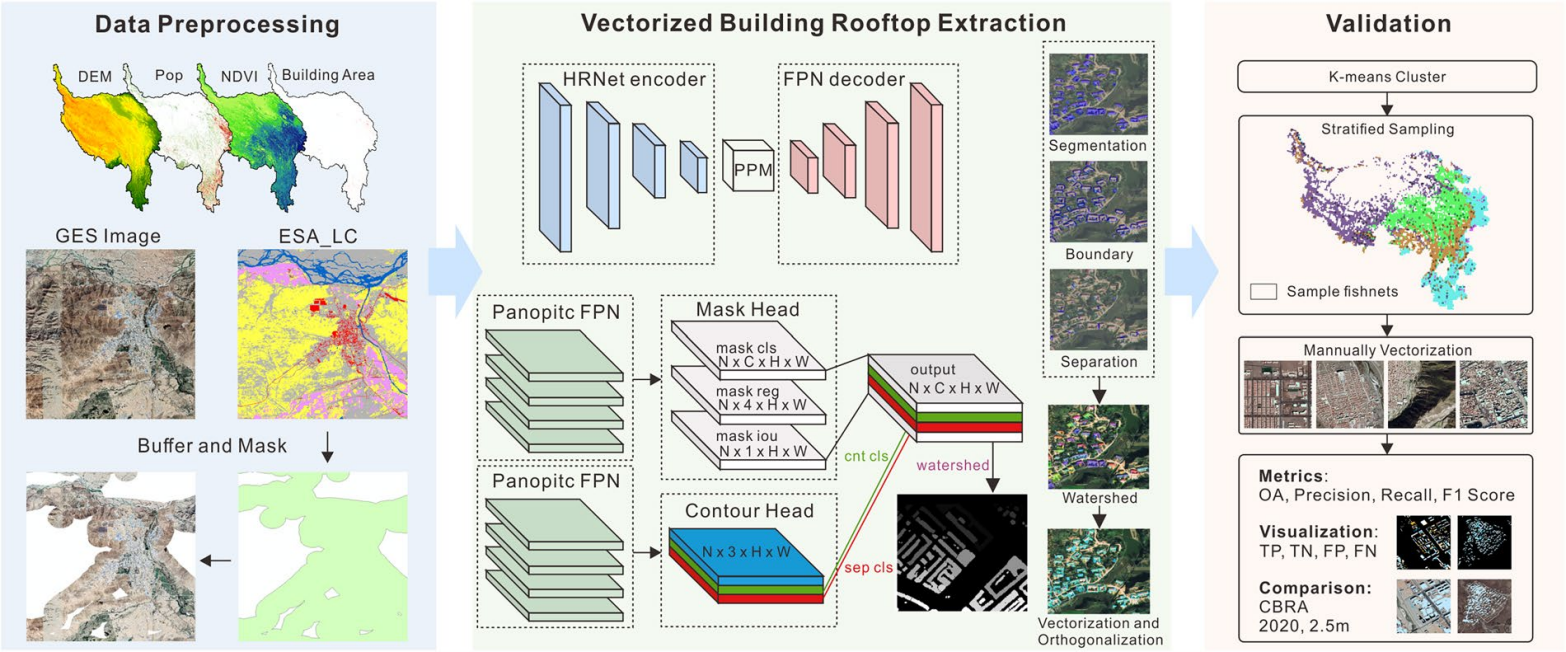
The framework for building rooftop extraction and validation.

In this study, we utilized building extraction algorithms from the AI Earth platform to generate a vectorized building rooftop dataset for the Qinghai-Tibetan Plateau and its neighboring region in China (Fig. 1). The principal components of our framework included: (1) satellite data and auxiliary data preparation and preprocessing; (2) vectorized building rooftop extraction using AI Earth platform; (3) validation using manually vectorized rooftop data.

### Study area

**Fig. 2:**
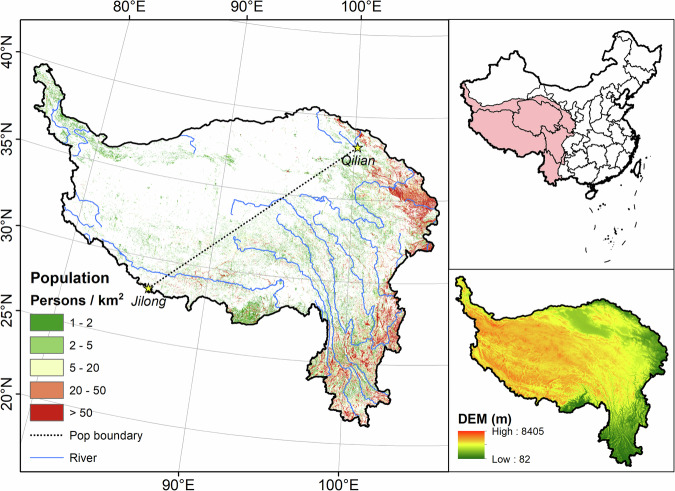
Study area (population data are from LandScan; DEM is from Resource and Environment Science and Data Centre).

This study mainly focused on the Qinghai-Tibetan Plateau and its neighboring regions in southwestern China under the general framework of the second Tibetan Plateau Scientific Expedition and Research Program^43^; geographically, it includes the Tibetan Autonomous Region, Qinghai Provinces, western Yunnan Province, western Sichuan Province, southwestern Gansu Province, and southern Xinjiang Autonomous Region. The average elevation of the study area is 4000-m above sea level. The distribution of population and buildings in the study area is highly influenced by elevation and climate (Fig. 2), mainly concentrating east of the line from Jilong County in Tibet to Qilian County in Qinghai^44^; in the east, they distribute densely in the plain areas on the eastern edge of the region, including the river valleys in Yunnan Province, western Sichuan Province, and the Xining-Lanzhou Yellow River Basin. In the plateau surface west of the line, population and buildings are mostly distributed with limited agro-pastoral areas along the major river valleys, and along road traffic corridors.

### Satellite imagery

Open-access high-resolution satellite image data were obtained from Rivermap Co. (http://www.rivermap.cn/index.html), which were obtained from Google Earth’s integration of satellite imagery and aerial data. Among them, the satellite imagery mainly comes from DigitalGlobe’s QuickBird and WorldView commercial satellites, and the aerial photography is sourced from BlueSky in the UK and Sanbornin the US^45^. For each location, there were collections of multiple imageries with resolutions of up to 0.15 m in localized areas. Such data integration has been widely used for object recognition in complex scenes^46–49^ and has the potential for large-scale high-resolution mapping of object types^50^.

**Fig. 3:**
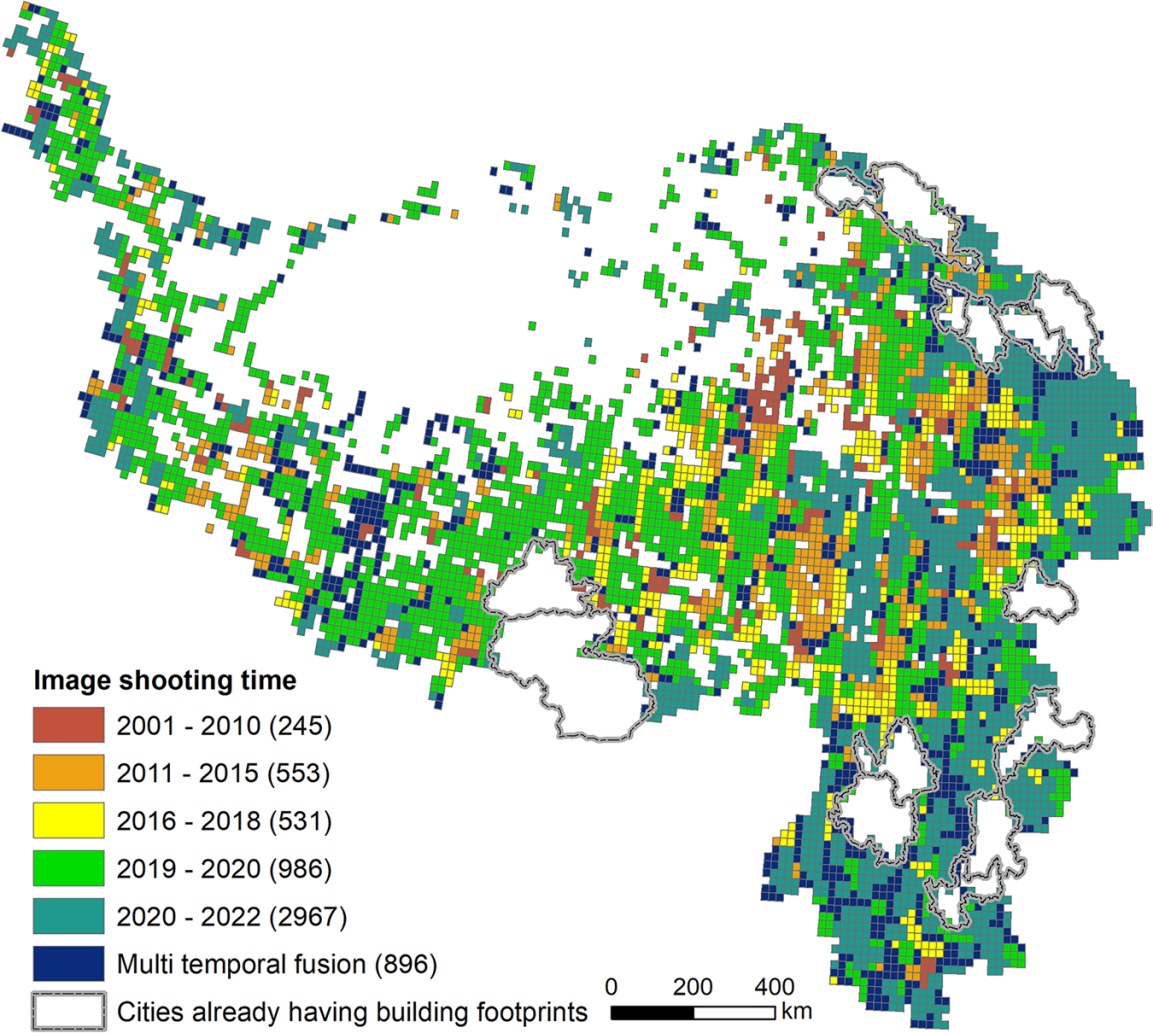
Fishnets for image download and image shooting time.

The total area of our study area is 3.06 million km^2^, and the estimated size of the satellite image data is 29.4 TB, with a spatial resolution of 0.6 m. Considering these scales, 0.175° × 0.175° fishnets were created for our study area to enable smaller-size packages for download, with a total of 10,033 fishnets used to cover the whole study area. The actual number of fishnets downloaded was smaller at 5921 for two reasons. First, as a large part of the study area comprises non-human residential areas where buildings do not exist, we arbitrarily excluded fishnets without any built-up area pixels from the ESA World Cover product. Second, cities/prefectures whose vectorized rooftop data (i.e., Lhasa, Shannan, Kunming, Xining, Haidong, Zhangye, Baiyin, Lanzhou, Chengdu, Dali, Lijiang, Kunming, Zhaotong, and Yuxi) have been retrieved in the vectorized rooftop area data for 90 cities in China^13^ were also excluded. The images were downloaded during September 2022 and January 2023, with a resolution of 0.6 m, a single frame size of ca. 3 GB, and a total size of 2.72 TB. Images downloaded were mainly taken from 2019 to 2021, but images from some remote areas may have been taken as early as 2001 (Fig. 3).

### Auxiliary data

Our auxiliary data included high-resolution land cover maps, digital elevation model (DEM), normalized difference vegetation index (NDVI), and population distribution data for subsequent cluster analysis for the purpose of sampling (Table 1).

**Table 1:** List of data used to generate and valid our datasets.

Source Dataset	Spatial-temporal Information	Description
High-resolution remote sensing image^64^	0.6 m	Obtained from Google Earth
2020 ESA World Cover^65^	10 m	Land cover data developed based on both Sentinel-1 and Sentinel-2 data with a global overall accuracy of about 75%
China National 1 km DEM (based on SRTM)^66^	1 km	Resampled from 30 m SRTM V4.1
LandScan Population^67^	1 km, 2021	Population distribution data based on high-resolution imagery exploitation, and a multi-variable asymmetric modeling approach
China Annual 1 km NDVI^68^	1 km, 2021	Based on the MODIS 16-day 250 m EVI product (MOD13Q1.061), synthesized using the maximum value synthesis method and then resampled to 1 km
China Building Rooftop Area (CBRA)^69^	2.5 m, 2021	Generated based on Sentinel-2 and deep learning algorithms with OA of 83%

For land cover and built-up area, ESA’s World Cover product from Zanaga *et al*.^17^ was obtained from Zenodo (https://zenodo.org/records/5571936). The product was generated based on Sentinel 1 and Sentinel 2 satellite imagery for the entire year of 2020 and a sample of 141,000 unique locations distributed around the world, trained with the random forest algorithm, to represent global land cover in 2020; it has an advantage in representing fine-scale landscape elements (e.g., built-up areas and complex agricultural landscapes), as it considers a relatively small minimum mapping unit^51^. The “built-up” category in the dataset refers to land covered by buildings, roads, and other man-made structures (e.g., railroads) but excluding urban green spaces (e.g., parks and sports facilities), landfill deposits, and mining sites^17^.

The 1-km DEM data and NDVI data were obtained from the Resource and Environment Science and Data Centre, Institute of Geoscience and Resources, Chinese Academy of Sciences (https://www.resdc.cn/Default.aspx). The DEM data are resampled from the latest SRTM V4.1 data (https://www.resdc.cn/data.aspx?DATAID=123), and the NDVI data are mosaiced based on SPOT/VEGETATION PROBA-V 1-km products (http://www.vito-eodata.be). We chose the NDVI data of 2021 to represent vegetation on the Qinghai-Tibetan Plateau (https://www.resdc.cn/DOI/DOI.aspx?DOIID=49). Population density data were obtained from the LandScan database (https://landscan.ornl.gov/) for global vital statistics analysis developed by the U.S. Department of Energy’s Oak Ridge National Laboratory and provided by East View Cartographic (https://geospatial.com/); these data are generated by combining geospatial science, remote sensing technology, and machine learning algorithms, representing one of the most accurate and reliable global population dynamic statistical analysis databases based on geographic location, with superior resolution at 1 km^52^. We used the population distribution in 2021 for subsequent cluster analysis.

### Data pre-processing

The distribution of population and buildings is scattered for most of our study area, with over 99.5% of the study area categorized as non-built-up areas according to the ESA’s World Cover product^17^. To expediate our extraction, masks of potential building distribution area were first generated for each 0.175° × 0.175° fishnet before extraction. Based on the “Built-up” category in the World Cover data, a 1-km buffer zone surrounding each built-up area pixel was generated as the potential building distribution area. After testing several buffer zone widths, we found that a width of 1 km could accommodate 96% of the building pixels reported in the CBRA products of 2020. The mask enabled us to exclude 86% of the total area of the downloaded imageries, consequently saving substantial computational time. We analyzed the buffer using an overlay with the generated fishnets to exclude nets that did not contain buildings. The images were then cropped again with buffers, and then the building extraction algorithm was applied to the cropped images.

### Vectorized building rooftop extraction

The vectorized building rooftop extraction algorithm used in this study is from the AliCloud AI Earth platform (https://engine-aiearth.aliyun.com/#/), which combines a deep learning–based segmentation method with a watershed-based segmentation method to construct a building instance segmentation framework - double decoder for watershed segmentation. This algorithm adds a boundary segmentation task to the semantic segmentation task, and uses the watershed algorithm to preprocess the prediction results of the two tasks during prediction, obtaining the final building extraction result. The PointRend neural network proposed by Kirillov *et al*.^53^ is used first^53^, which treats image segmentation as a rendering problem and employs an iterative segmentation algorithm that selectively samples non-uniform points for accurate segmentation, as more stable and accurate seed points learned by the neural network can provide finely tuned semantic segmentation models for key structures and features of the building. Subsequently, a flexible watershed segmentation is used for post-processing^54,55^, which is able to adapt to objects with different morphologies and features. The algorithm achieves a counting accuracy of >90% and an area estimation accuracy of >85% in validation testing based on manual vectorized samples in different regions of China, winning second place in the all-weather SAR image building segmentation competition SpaceNet6. Compared to Mask Region-CNN, the algorithm improves the mean average precision by 11 percentage points^56^.

## Data Records

**Fig. 4:**
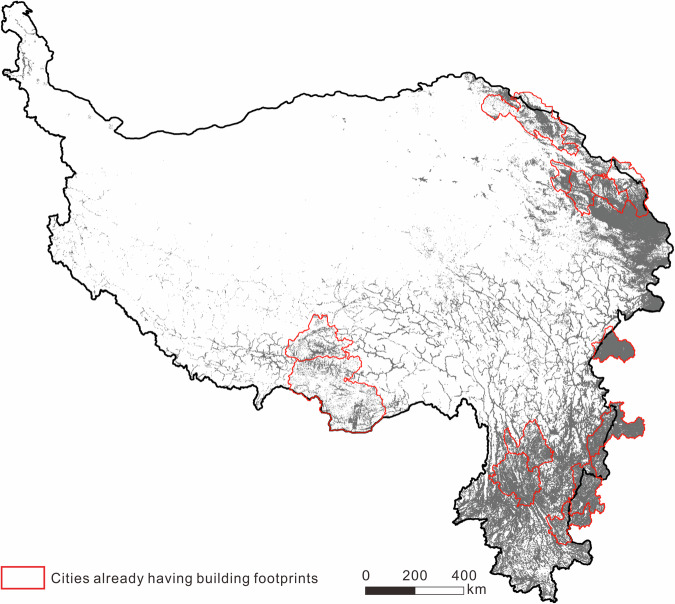
Extraction results.

The vectorized building rooftop extraction algorithm used in this study can be called on the AI Earth platform (https://engine-aiearth.aliyun.com/#/). Our dataset is available from the National Tibetan Plateau Data Centre, which can be accessed at 10.11888/RemoteSen.tpdc.301170^57^. All data are obtained using the GCS_ WGS_ 1984 coordinate system and packaged into.rar files (Table 2). The generated AI-based building contour data (Fig. 4) is arranged on province level according to their name, including Gansu, Guizhou, Qinghai, Sichuan, Xinjiang, Xizang, and Yunnan. In addition, image data of the corresponding 250-km^2^ grid and manually drawn verification data from original sources were also uploaded in ‘image_1km.rar’ and ‘test_1km.rar’. The year of image acquisition in Fig. 3 is in ‘image_time.rar’.

**Table 2:** Information of files in generated datasets.

Filename	Description	File type
image_1km	High resolution image of 250 1 km^2^ grids for validation	GeoTIFF
image_time	Acquisition time of image in each fishnet	ESRI Shapefile
test_1km	Manually vectorized building contours from original sources in 250 validation grids	ESRI Shapefile
gansu, guizhou, qinghai, sichuan, xinjiang, xizang, yunnan	AI based building contour extraction results on province level	ESRI Shapefile

## Technical Validation

### Validation data preparation based on stratified sampling

**Fig. 5:**
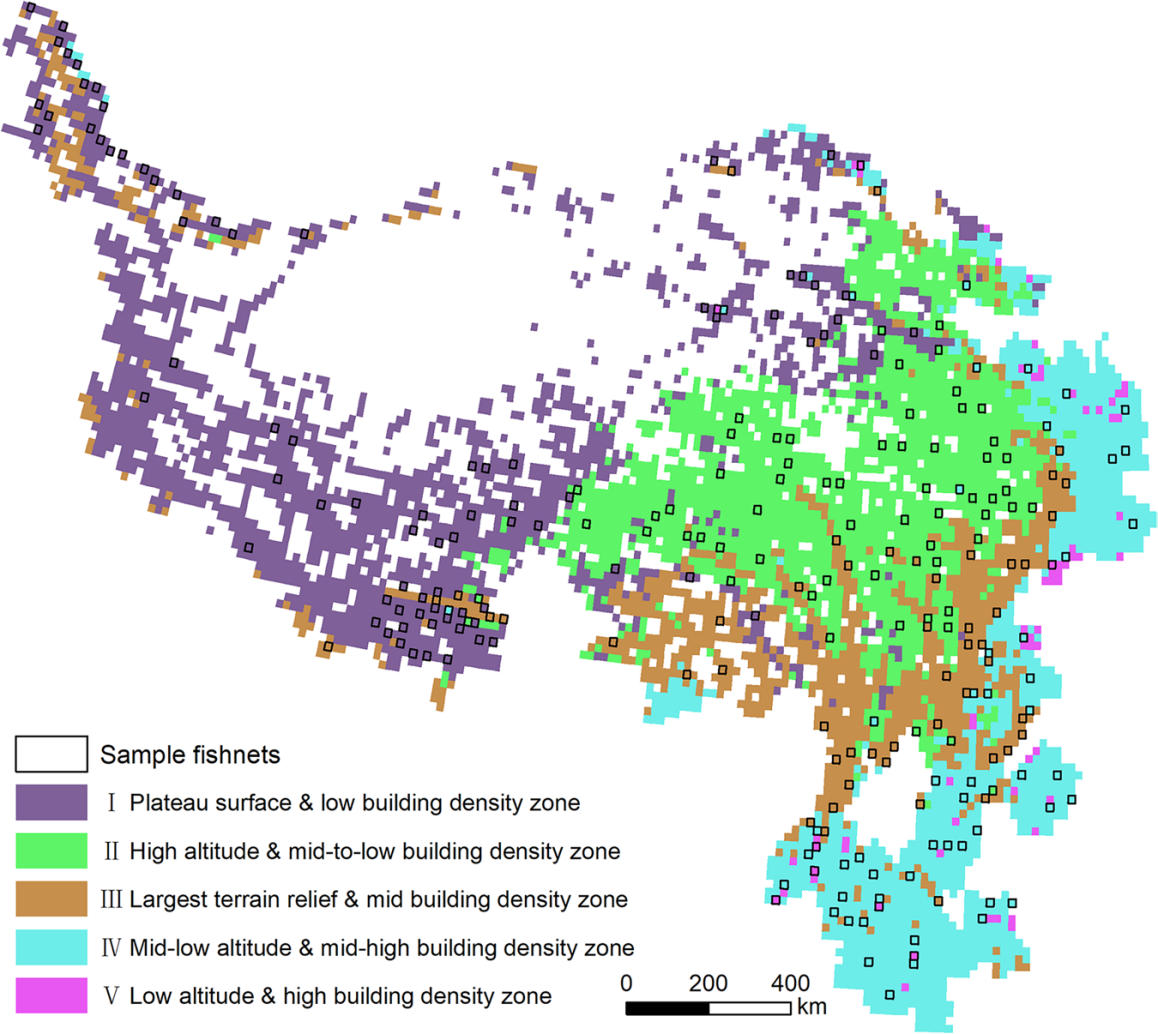
Geographical zoning map and fishnets for creating validation dataset.

Manually building rooftop vectorization was conducted to derive “ground-truth” building rooftop data for validation purposes. Due to the vast area of our study area, as well as substantial regional differences in terms of elevation, landform, vegetation type, and building type, we used a stratified sampling approach instead of random sampling to obtain a balanced sample in terms of different sub-regions. We performed K-means clustering^58^ on the 5921 fishnets based on five indicators: built-up area, mean NDVI, population density, mean elevation, and standard deviation of elevation. K-means clustering is a clustering algorithm based on Euclidean distance, in which the closer the distance between the characteristics of two targets, the greater the similarity. After standardizing the data by subtracting the mean and dividing by the standard deviation, the elbow method was used to select the most suitable number of categories^59^. During the process, when the number of categories was increased to five, the rate of decrease of the sum of squared errors declined rapidly. Therefore, we clustered the 5921 fishnets into five categories for subsequent analyses (Table 3, Fig. 5).

**Table 3:** Statistics for different geographical clusters.

Cluster	Count of fishnets	Average DEM	Standard deviation of DEM	Average NDVI	Average Population	Build Area	Sample fishnets selected
I	1947	3767	220	2061	3717	58820	81
II	1670	3992	230	6548	12818	107045	60
III	1045	2933	587	5740	15867	153031	50
IV	1193	1860	247	6110	82823	700123	51
V	66	1589	221	5313	188467	2384441	8

Cluster I (plateau surface & low building density zone) covers a large area on the plateau surface of the Qinghai-Tibetan Plateau—with the widest area and the flattest terrain—and is characterized by high elevation, low population, low vegetation cover, and low built-up area. Fishnets in Cluster II (high altitude & mid-to-low building density zone) are mainly located at the border regions of Qinghai, Sichuan, and Tibet, and are mostly covered with alpine meadows and shrubs, and mainly the headwaters of large rivers in China. However, the climate conditions of Cluster II are relatively harsh, resulting in a relatively sparse population and buildings here. Fishnets in Cluster III (largest terrain relief & mid building density zone) have the greatest variability in elevation, located mainly in the topographic transition zone around the plateau. Cluster IV (mid-low altitude & mid-high building density zone) mainly includes Gansu and Yunnan provinces in the eastern part of the Qinghai-Tibetan Plateau, with relatively low elevation, lush vegetation, and dense population and buildings. Cluster V (low altitude & high building density zone) has the smallest number of fishnets but the most urban area, the lowest average elevation, and the highest population and building densities.

A total of 250 fishnets were then selected based on the division of clusters; within each fishnet, a 1-km^2^ grid was used to prepare ground truth data for accuracy validation, which yielded a sampling rate of 4.22% (250/5921 fishnets) or 0.093% (250 km^2^/267904 km^2^ buffer mask). The number of the sampling fishnets selected for each cluster is proportionate to its total size. Within each cluster, we give priority to fishnets with large built-up area in ESA’s World Cover product. During this process, we also attempted to avoid selecting neighboring fishnets so that the sample could guarantee a better spatial coverage. The final distribution of selected fishnets is shown in Fig. 5. Each sampled fishnet was then divided into 1-km^2^ grids, and the grid with the largest built-up area according to ESA’s World Cover product was selected for manual building rooftop vectorization. Finally, we obtained a total of 149,035 manually outlined buildings with a total area of 24.65 km^2^.

### Validation result

We used a quantitative approach^12^ to validate our extraction results, referring to a multi-criteria hierarchical evaluation system for evaluating buildings extracted based on remote sensing (Zeng *et al*.^60^). Based on the manually vectorized building rooftop data, the match rate metrics (Table 4) of the 250 × 1 km^2^ grids, including overall accuracy (OA), precision, recall, and F1 score for precision evaluation^61^, were computed based on the confusion matrix^62^.

**Table 4:** Evaluation metrics.

Metric	Description
True Positive (TP)	Correctly extracted building roof plots
False Positive (FP)	Background misclassified as a sample of a building roof
True Negative (TN)	Parcels correctly categorized as background
False Negative (FN)	Roofs of buildings misclassified as background
Overall Accuracy (OA, %)	(TP + TN)/(TP + FP + TN + FN) Proportion of correctly recognized buildings and background
User Accuracy/Precision (%)	TP/(TP + FP) Proportion of real buildings in the results
Producer Accuracy/Recall (%)	TP/(TP + FN) Proportion of extracted buildings in validation data
F1 Score (%)	2 × TP/(2 × TP + FP + FN) Weighted average of the precision and the recall

The OA of our result is 87%, indicating that our dataset has high credibility in extracting buildings and excluding backgrounds (Table 5). However, a precision score of 50.1% indicates that approximately half of building rooftop areas extracted are incorrect, which is mainly due to false prediction of building spacing in areas of high building density (e.g., in high-density built-up areas where the spacing between buildings is small). The recall of our results is approximately 92%, which means that manually vectorized building roofs can be mostly extracted by the algorithm. Our results were slightly better than CBRA^12^ and the vectorized rooftop area data for 90 cities in China^13^. Compared with our result, based on our validation dataset, CBRA has a comparable OA but relatively small precision, recall, and F1 score. The vectorized rooftop area data for 90 cities in China were reported to have an OA of 83.4% and a recall of 79.0%^12^, which may be due to the image semantic segmentation model it used lacking a specialized extraction module.

**Table 5:** Performance metrics for building rooftop extraction results.

Cluster	Grids	OA		Precision		Recall		F1 Score	
		Our	CBRA	Our	CBRA	Our	CBRA	Our	CBRA
I	81	94.6	90.2	52.7	22.7	78.8	36.7	60.6	24.6
II	60	87.9	82.2	48.6	30.7	87.5	49.4	61.6	36.2
III	50	88.2	85.0	48.6	36.8	91.8	59.1	62.5	44.4
IV	51	75.7	72.0	52.5	47.3	93.8	72.1	66.7	56.5
V	8	72.0	66.8	52.6	48.4	96.3	87.4	67.5	61.8
Whole Grids	250	87.0	82.7	50.1	40.2	91.9	67.2	64.8	50.3

The performance of our extraction differed by fishnet clusters, suggesting the challenges of distinguishing rooftop from environmental background, and proving the reasoning of adopting stratified sampling in validation (Table 5). There are pronounced differences in the OA and recall between clusters. In general, OA decreases but recall decrease with the decrease of average altitude and the increase of built-up area. However, the difference in precision between clusters is relatively small, indicating that the proportion of real buildings in the extracted results of each cluster is relatively close.

**Fig. 6:**
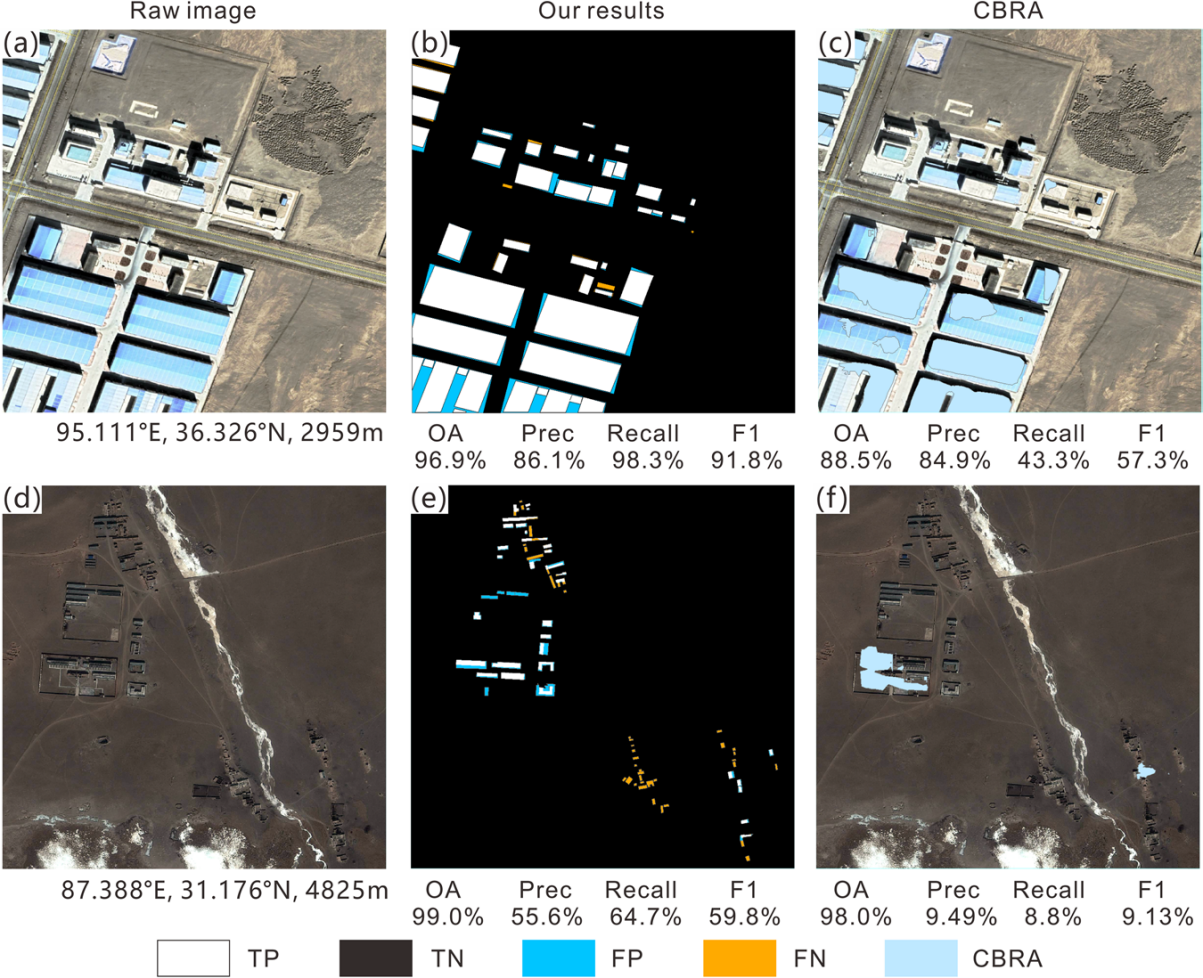
Accuracy evaluation for Cluster I (**a,d**: raw image; **b,e**: our results; **c,f**: CBRA).

**Fig. 7:**
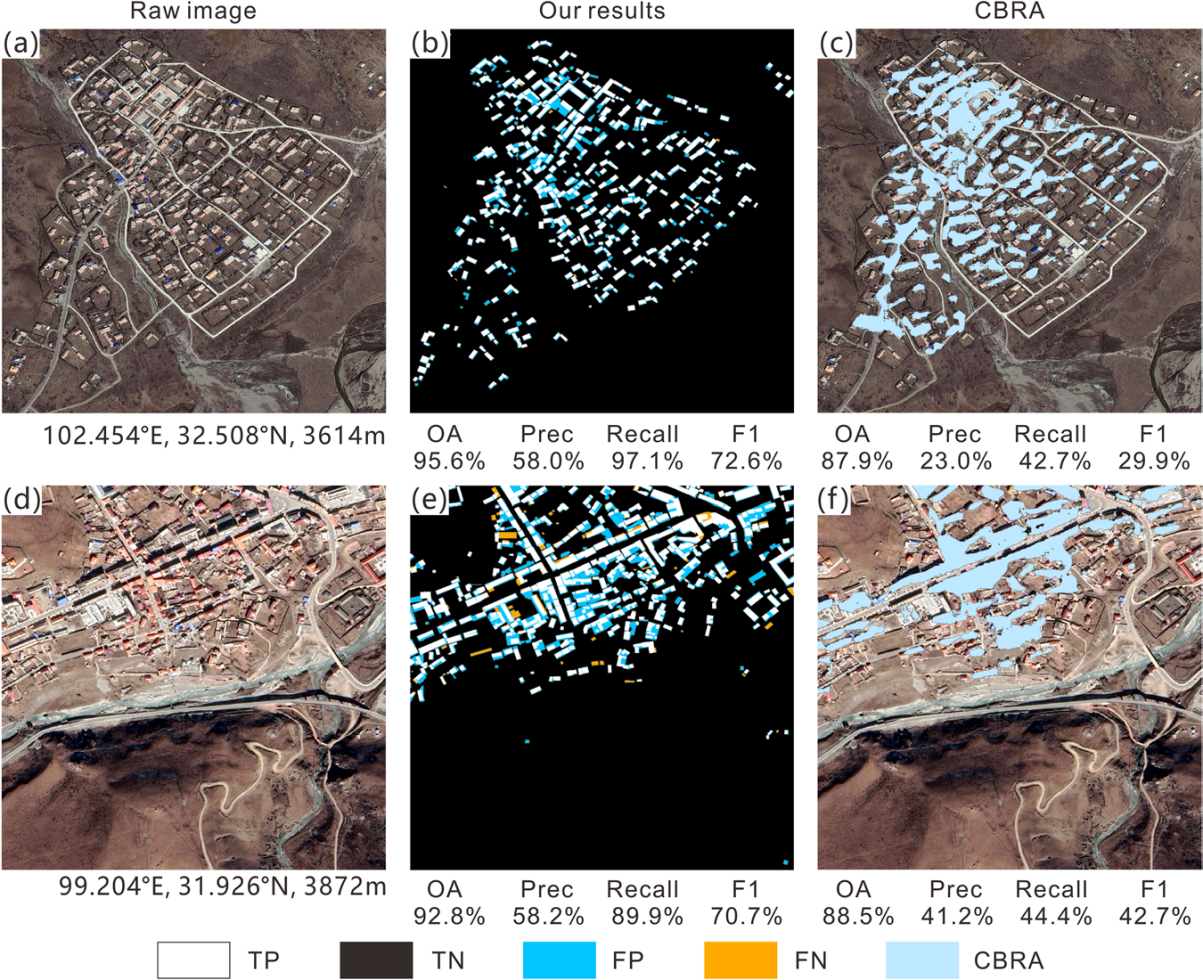
Accuracy evaluation for Cluster II (**a,d**: raw image; **b,e**: our results; **c,f**: CBRA).

**Fig. 8:**
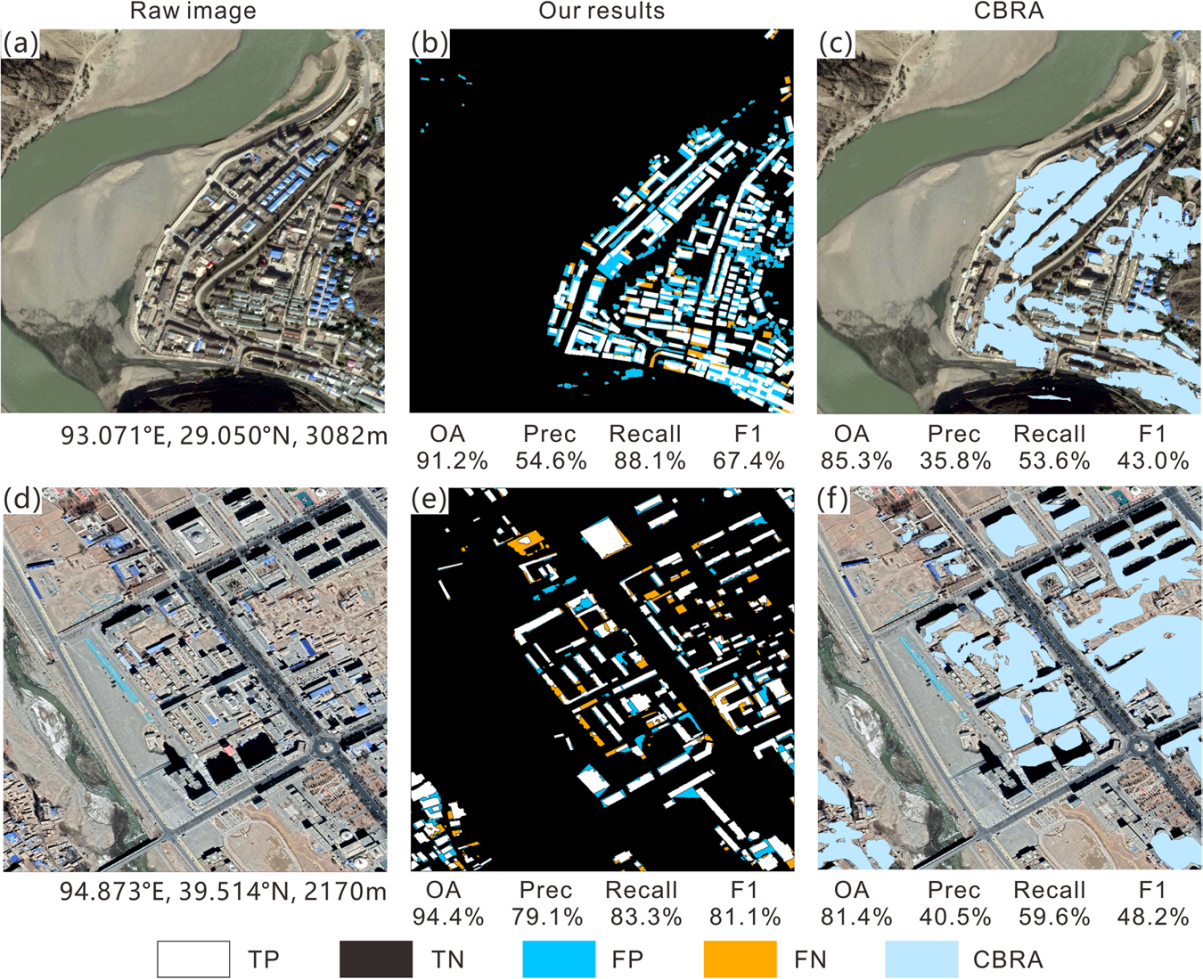
Accuracy evaluation for Cluster III (**a,d**: raw image; **b,e**: our results; **c,f**: CBRA).

**Fig. 9:**
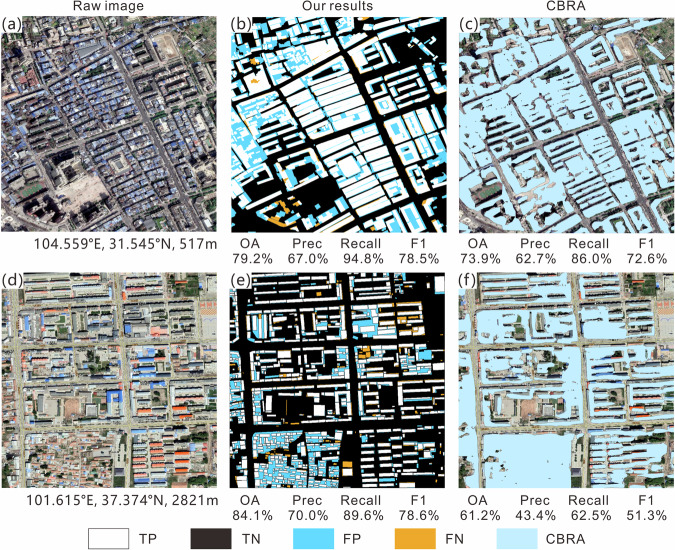
Accuracy evaluation for Cluster IV (**a,d**: raw image; **b,e**: our results; **c,f**: CBRA).

**Fig. 10:**
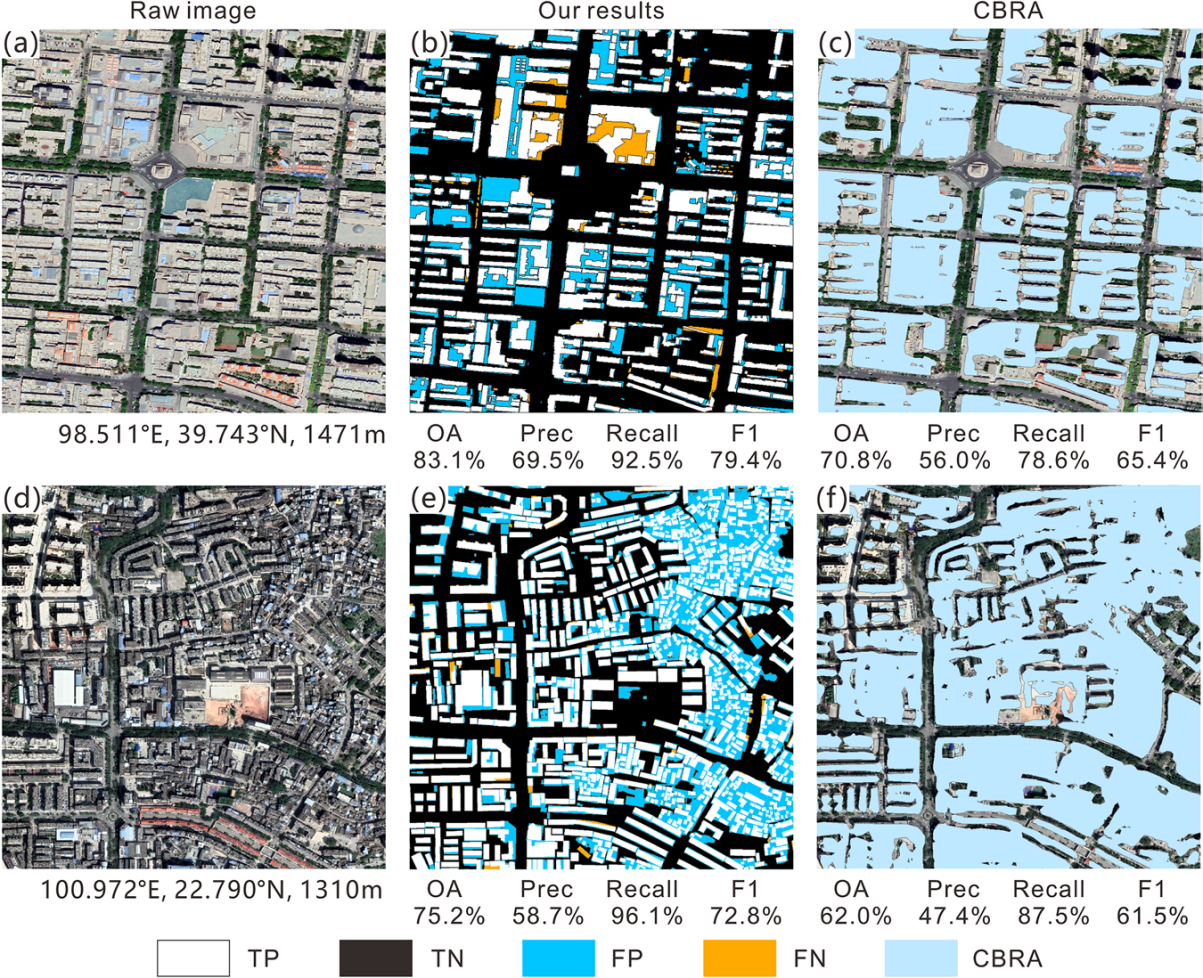
Accuracy evaluation for Cluster V (**a,d**: raw image; **b,e**: our results; **c,f**: CBRA).

To further understand the challenges of extraction, the visualization results of elements in the confusion matrix (TP, TN, FP, and FN) for different clusters are shown in Figs. 6–10; each sub-image corresponds to a sampled 1 km^2^ grid, with the original image on the left, the extracted results from this study in the middle, and the results of the CBRA product on the right. Elements in the confusion matrix—TP, TN, FP, and FN—correspond to correct building, correct background, misidentified building, and unidentified building in the legend, respectively. Validation metrices are also supplied below their corresponding results.

Cluster I mainly covers the plateau surface area of Qinghai-Tibetan Plateau, which is the largest and flattest among the five clusters, with sparse vegetation and widespread bare land. The buildings in this cluster mainly exhibit sparse distribution on a large scale and dense distribution locally (Fig. 6a); it has the highest OA (94.6%) and precision (52.7%) among the five clusters, but the lowest recall (78.8%) and F1 score (60.6%). The low recall indicates that there is still a considerable portion of buildings in the cluster that have not been extracted by the algorithm. However, the high OA is maintained in this cluster due to the small area of buildings relative to the large area of background. The roofs in densely populated local places are mainly white and blue, including some large industrial plants (Fig. 6a). Their difference from bare land gives the relevant 1-km grids high extraction accuracy (approximately 100% OA and recall). The houses in sparsely distributed areas are mostly single-story residential buildings, with gray-black roofs that are less distinguishable from the surrounding bare land, resulting in FN (e.g., the scattered buildings in the bottom-right corner of Fig. 6d). In this cluster, CBRA has a comparable OA but relatively small recall, precision, and F1 score according to our validation dataset; its high OA is also due to its correct recognition of large areas of background.

Cluster II is mainly located in the high-altitude area in eastern Qinghai-Tibetan Plateau; its population and built-up area are slightly larger than those of cluster I, with a higher number of settlements, but still the general distribution of buildings is relatively sparse. This cluster has a relatively higher OA (87.9%) and a relatively lower precision (48.6%) among the five clusters (Table 5); it has many red-tiled and blue-roofed masonry buildings, making it easier to distinguish the buildings from the brown bare ground. This results in a remarkable improvement in recall (87.5%), and the issue of missing building blocks (FN) is also relieved, as compared to cluster I. However, as the number of buildings increases, the amount of FP also begins to rise. The CBRA product has a good recall (49.4%) in this cluster but experiences difficulty in extracting individual buildings (Fig. 7).

Cluster III is a transitional area from high altitude mountainous plateaus to low altitude hilly plains. As altitude decreases, population density and built-up area further increase. It has the largest terrain relief among the five clusters. The buildings in this cluster are mainly concentrated in low mountain and valley areas and are distributed along rivers and contour lines (Fig. 8a). At the same time, a certain number of high-rise residential buildings appear in the cluster, whose rooftops are mostly gray with obvious edges, and a relatively large distance between them (Fig. 8d). Therefore, the algorithm is more precise when extracting their rooftop contours compared to other buildings, yielding a high recall for this cluster (91.8%). However, shadows caused by high-rise buildings and mountains resulted in increased FNs, and the background pixels at the shadow edges were misidentified by the algorithm, leading to some FPs. CBRA also has better validation metrics in this cluster compared to clusters I and II, as it can identify the buildings in the main densely populated areas. However, it also is negatively impacted by building shadows (FP, bottom of Fig. 8a).

Cluster IV is mainly located in valley and small-plain areas with lower elevations and flatter terrain around the plateau. It has a larger average built-up area, more population, and more large-scale individual buildings than clusters I–III, and the arrangement of buildings in this cluster is more orderly (Fig. 9a,d). Based on this context, the OA (75.7%) of this cluster is smaller, whereas the precision (52.5%), recall (93.8%), and F1 score (66.7%) are all higher to varying degrees compared to clusters I–III. For buildings with clear boundaries and large rooftop areas, our results maintain their integrity and sharp edges. Our results well capture contiguous building areas, and the extraction of external envelope lines is very successful. However, our method struggled to distinguish densely connected buildings, such as apartment buildings, whose building spacing is often small. This difficulty led to blob-like segmentation results. For example, in the densely populated area shown in Fig. 9d, the open spaces between buildings have similar spectral features to the buildings, and the distance between adjacent buildings is small. Although this phenomenon also exists in CBRA, relatively speaking, our results have a lower proportion of FP by identifying some small roads in dense building areas (Fig. 9d). At the same time, both products did not mistakenly identify the main road as a building, indicating that they can effectively distinguish the characteristic differences between roads and buildings^63^.

Cluster V mainly comprises densely populated urban areas with low and flat terrain around the plateau. Its OA (76.8%) is the lowest among all clusters, mainly due to the increase in FP and the decrease in TN (Fig. 10e). However, this cluster has the highest recall (96.3%) and F1 score (67.5%) among the five clusters. The high recall indicates that most of the buildings in the cluster can be extracted by the algorithm (Fig. 10b,e), which may be because it has been well trained by the AI Earth platform based on urban areas with high building density. At the same time, the urbanization level of this cluster is higher, and there are fewer dense and small scattered buildings that are difficult to distinguish from the background, which slightly improve precision (0.4%) and F1 score (1.4%) compared with cluster IV. CBRA is similar to our results, but there is also a problem of FN in areas of high building density. For the special case of small buildings at the top of a large-area building (Fig. 10a,b), our results consider the bottom building as the background value, whereas CBRA can fully extract the entire building.

## Usage Notes

The high-resolution building rooftop prints extracted based on AI Earth building rooftop extraction algorithm in the Qinghai-Tibetan Plateau and its neighboring region is suitable for research on large-scale building distribution, spatial structure, urbanization process, and human activity intensity. In a complex and ecologically sensitive region like the Qinghai-Tibetan Plateau, such building data can provide important support for urban and rural planning, infrastructure assessment, and research on human land relationships. In addition, it can provide building exposure data foundation for disaster risk assessment and ecological protection planning.

Our dataset also offers original data of 250 × 1 km^2^ manually vectorized building rooftop data. Although the coverage is small as compared to the area of the study area, it is very suitable as benchmark data to evaluate the accuracy and stability of automated building extraction algorithms due to its high accuracy and controllable errors. In addition, it can also be used for more detailed research tasks such as building density analysis, micro scale urban structure research, and architectural style recognition. If further combined with ground measurements, it can serve as an important data foundation for architectural research in high-altitude special areas.

Our method to some extent balances the convenience of data acquisition and the efficiency of automated processing, but there are still some limitations that cannot be ignored from multiple perspectives.

Firstly, from the perspective of data sources, although the remote sensing images provided by Google Earth have high resolution, they enable us to obtain data covering the entire research area at a lower cost. However, due to the large research area, these images are not uniform, and there are differences in clarity, lighting conditions, and shooting angles among images obtained from different regions and at different times. Some images of fishnet can be traced back to 2001, with low resolution, resulting in less detailed spatial information and greatly reducing extraction accuracy. In the future, this problem can be solved by replacing these images with newer ones.

Secondly, from an algorithmic perspective, the building extraction algorithm of Alibaba Cloud AI Earth platform performs well overall in the Qinghai-Tibet Plateau scene. However, due to the influence of easily confused backgrounds, there are still adhesion phenomena in densely built areas, especially in scenes with Tibetan style buildings or small settlements on the plateau that are dense but strongly obscured, and the accuracy of building contour extraction will decrease. In the future, deep-learning-based edge detection modules can be further added to the extraction algorithm to enhance the extraction of architectural form features.

Lastly, as for the extraction results, due to the use of masks from ESA’s 10 m land cover products, there may be a small number of scattered building areas with built-up areas less than 100m^2^ that are missing. In addition, historical images can be used to generate a dynamic distribution of buildings over many years in the future to provide more detailed building information.

## Data Availability

No custom code was used to generate or process the first vectorized building rooftop prints of the Qinghai-Tibetan Plateau and its neighboring regions. The website of AI earth platform is https://engine-aiearth.aliyun.com/. The software used in the technical validation of our dataset was ArcMap version 10.8.
